# Acute pancreatitis risk in the diagnosis and management of inflammatory bowel disease: A critical focus

**DOI:** 10.1515/med-2025-1189

**Published:** 2025-05-22

**Authors:** Feibo Zheng, Jinan Li, Lina Ma, Yu Zhang, Zhengwei Tu, Yunfeng Cui

**Affiliations:** Department of Hepatobiliary and Pancreatic Surgery, Department of Surgery, Tianjin Nankai Hospital, Nankai Clinical School of Medicine, Tianjin Medical University, Tianjin Key Laboratory of Acute Abdomen Disease Associated Organ Injury and ITCWM Repair, Institute of Integrative Medicine for Acute Abdominal Diseases, Tianjin, 300100, China; Department of Surgery, Tianjin Occupational Diseases Precaution and Therapeutic Hospital, Tianjin, 300011, China; Department of General Surgery, The First Affiliated Hospital of Jinzhou Medical University, Jinzhou, Liaoning, 121000, China; Department of Health Management, Tianjin Hospital, Tianjin, 300211, China

**Keywords:** pancreatitis, autoimmune diseases, Mendelian randomization, GWAS, bidirectional

## Abstract

**Objective:**

We explored the causal relationship between pancreatitis and various autoimmune diseases using bidirectional Mendelian randomization (MR).

**Methods:**

We collected genome-wide association study summary data for four pancreatitis types and five autoimmune diseases to conduct our bidirectional MR analysis. The primary analysis was performed using the inverse variance weighted (IVW) method, complemented by MR Egger, weighted median, and weighted mode methods. Sensitivity analyses included Cochran’s *Q* test for heterogeneity, MR-Egger regression for pleiotropy, and MR-PRESSO and leave-one-out analyses for outliers.

**Results:**

The result of IVW revealed a significant association between genetically predicted inflammatory bowel disease (IBD) and an increased risk of acute pancreatitis (AP) (odds ratio [OR] = 1.07, 95% confidence interval [CI] = 1.03–1.12, *P* = 0.0015). Subsequent analyses further confirmed this association in IBD subtypes, with genetically predicted ulcerative colitis (UC) and Crohn’s disease (CD) also showing increased risks of AP (UC: OR = 1.07, 95% CI = 1.02–1.13, *P* = 0.01; CD: OR = 1.05, 95% CI = 1–1.09, *P* = 0.03), affirming IBD as a risk factor for pancreatitis. Reverse analysis ruled out reverse causality and did not find a causal relationship between other immune diseases and pancreatitis.

**Conclusion:**

These findings suggest that pancreatitis in IBD patients may arise from the disease itself, necessitating increased vigilance for AP during diagnosis and treatment.

## 1 Introduction

Pancreatitis is a common digestive disease worldwide and a leading cause of emergency gastrointestinal hospitalizations [1]. According to epidemiological data, in 2019, there were 2,814,972 cases of pancreatitis worldwide, resulting in 115,053 deaths [2]. In recent years, the incidence of pancreatitis has been steadily increasing, with a high mortality rate among severe cases and significant consumption of medical resources, becoming one of the major diseases that endanger people’s health and lives [2,3].

Retrospective clinical research has revealed that the onset and development of pancreatitis are strongly correlated with immunological factors and the immune system. Gastrointestinal lesions of autoimmune diseases can present as pancreatitis, and patients with this type often have a more severe condition. For example, acute pancreatitis (AP) more commonly occurs during the active phase of systemic lupus erythematosus (SLE) (89%) and the incidence of ascites, sepsis, and hypocalcemia in SLE-associated AP increases, with a mortality rate reaching over 30% [4,5]. Rheumatoid arthritis (RA) can increase the risk of acute/chronic pancreatitis (CP) [6]. Inflammatory bowel disease (IBD) patients often exhibit symptoms of pancreatitis, such as exocrine dysfunction, abnormal pancreatic ducts, and hyperamylasemia. Pancreatitis, especially type 2 autoimmune pancreatitis, is common among IBD patients [7]. Moreover, the incidence of AP has been reported to be higher in IBD patients than in the non-IBD population, with Crohn’s disease (CD) patients and ulcerative colitis (UC) patients, having a 4.1 and 2.6 times higher incidence of AP, respectively [8]. Type 1 diabetes (T1D) is an autoimmune disease where the immune system attacks the insulin-producing β cells, possibly accompanied by pancreatitis [9]. Studies indicated that autoimmune diseases could initiate or exacerbate pancreatitis by modulating immune-inflammatory responses, cytokines, lymphocyte function, and intestinal barrier function, leading to pancreatic tissue damage [10]. These clinical observational studies suggest a potential correlation between autoimmune diseases and pancreatitis. However, it is challenging to avoid confounders such as treatment approaches and medication use, which hinder the ability to establish a clear causal relationship between these diseases. In addition, reverse causality and the inability to establish a clear temporal relationship also affect the establishment of causality.

Mendelian randomization (MR) is a method for detecting potential causal relationships between exposure factors and disease outcomes, which has become increasingly popular in recent years [11]. Compared to traditional observational studies, MR utilizes genetic variations as instrumental variables (IVs). These genetic markers are randomly assorted at conception and remain largely unaffected by the environmental or lifestyle factors that typically confound observational research. By leveraging these genetic variations, MR can more reliably infer causality, avoiding the pitfalls of reverse causality. This aspect makes MR particularly valuable in providing robust evidence for the pathogenesis of diseases.

The present study focused on the potential causal relationships between various types of pancreatitis – including AP, CP, alcohol-induced AP, and alcohol-induced CP – and autoimmune diseases such as RA, SLE, IBD, CD, UC, and T1D. To clarify the directionality of the relationship between autoimmune diseases and pancreatitis, we hypothesize that autoimmune diseases may causally increase the risk of pancreatitis, while pancreatitis does not influence the risk of developing autoimmune diseases. This research aims to provide new biological diagnostic markers, treatment strategies, and a theoretical basis for further investigations into the mechanisms underlying the interaction between autoimmune diseases and pancreatitis.

## 2 Methods

### 2.1 Study design

**Figure 1:**
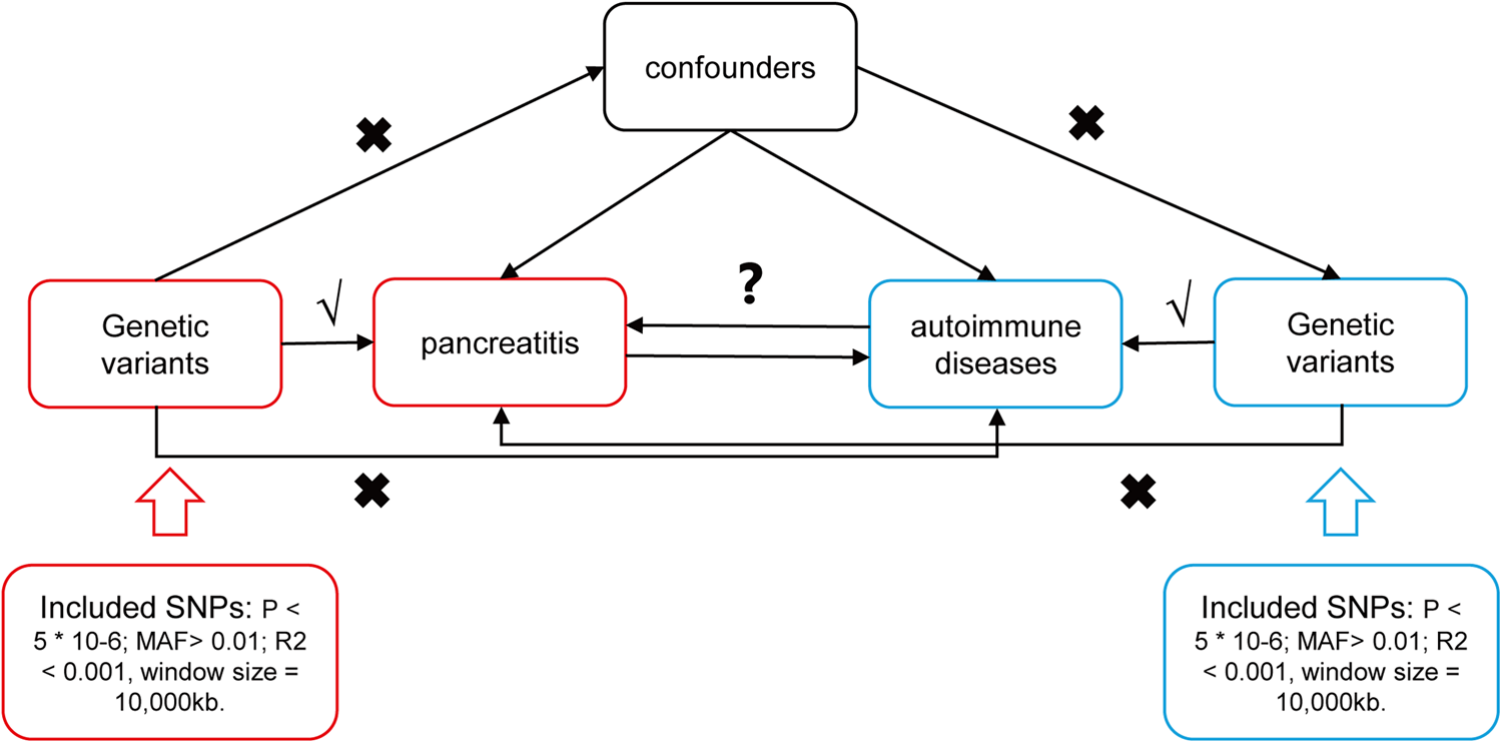
The experimental study design: Schematic representation of MR hypothesis.

Considering the findings above and the MR method, we downloaded genome-wide association study (GWAS) data for pancreatitis and autoimmune diseases. As shown in Figure 1, a bidirectional two-sample MR analysis was chosen to determine the causal association between pancreatitis and autoimmune diseases. Employing bidirectional MR strengthens the findings by ruling out reverse causality, thus providing robust insights into the nature of these interactions. MR operates under three critical assumptions: (1) the selected genetic IVs have a strong association with the exposure; (2) these genetic IVs are independent of any confounders that might influence both the exposure and the outcome; and (3) the effect of the genetic IVs on the outcome is exclusively mediated through the exposure, with no alternative biological pathways.

### 2.2 Data sources

GWAS summary data for four types of pancreatitis were obtained from the FINNGEN database (https://www.finngen.fi/en), with 16,380,428 single-nucleotide polymorphisms (SNPs). The FINNGEN project aims to study the genetic basis and risk factors of human diseases by utilizing nationwide health records and genetic data in Finland. Precisely, the samples for AP included 3,022 cases and 195,144 controls; CP contained 1,737 cases and 195,144 controls; AP induced by alcohol comprised 457 cases and 218,335 controls; and CP induced by alcohol included 977 cases and 217,815 controls. Autoimmune diseases included six diseases, with RA GWAS data obtained from 18 European studies [12], SLE [13] GWAS data from a cohort of Europeans in 2015, IBD (including CD and UC) [14] GWAS data from a European cohort in the UK IBD Genetics Consortium and UK10K Consortium, and T1D [15] GWAS data from a meta-analysis of studies on individuals from the UK and Sardinia. Details of the GWASs included in the MR are shown in Table S1.

### 2.3 IV selection

The IVs incorporated in the present study were required to meet the following criteria: (1) SNPs with a robust association to the exposure were identified from the exposure GWAS. Due to the stringent *P*-value threshold of 5 × 10^−8^ not yielding a sufficient number of IVs, a *P*-value of <5 × 10^−6^ was utilized to ensure adequate coverage and strength of the genetic instruments; (2) SNPs exhibiting a minor allele frequency >0.01 were chosen; (3) to mitigate the impact of linkage disequilibrium among SNPs, SNPs were selected based on the criteria of *R* ^2^ < 0.001, window size = 10,000 kb; if the selected IV was not present in the outcome summary data, an SNP with high LD (*R* ^2^ > 0.8) with the IV was sought as a proxy SNP for replacement; and (4) the *F* statistics for each SNP in the IV was calculated to assess the strength of the IV, to exclude the possibility of weak IV bias between the IV and the exposure factor, with the formula for *F* statistics being *F* = *R* ^2^ × (*N* – 2)/(1 – *R* ^2^), requiring *F* statistics >10.

### 2.4 MR analysis

The two-sample MR analysis between exposure and outcomes was conducted via the “TwoSampleMR” R package [16]. The primary method used for analysis was the inverse variance weighted (IVW) method, which assessed the causal link between exposure and outcome risks by calculating the odds ratios (OR) and their 95% confidence interval (CI). The IVW method was used as the primary tool for interpreting MR results, calculating the weighted average of effect sizes using the inverse variance of each SNP as weights. This analysis also employed the MR-Egger, weighted median, and weighted mode methods to test the robustness of the results. The MR-Egger method provides accurate causal effect estimates in the presence of pleiotropy bias by considering the existence of an intercept term. Based on the assumption that at least half of the IVs are valid, the weighted median method analyzes the causal link between exposure and outcome. Statistical significance is determined by a *P* of less than 0.05.

### 2.5 Sensitivity analysis

In MR investigations, sensitivity analysis was used to uncover potential issues with pleiotropy. Cochran’s *Q* test was employed for evaluating heterogeneity across IVs, with no significant heterogeneity acknowledged if the *P*-value of >0.05 [17]. Potential pleiotropy was detected via the MR-Egger regression method; an intercept near zero and a *P*-value of >0.05 were interpreted as indications of negligible pleiotropy bias [18]. Additionally, we harnessed the MR pleiotropy residual sum and outlier (MR-PRESSO) approach to pinpoint potential outlier SNPs (those with a *P*-value <0.05) and adjusted for pleiotropy bias by recalculating the causal estimate post-outlier removal [19].

## 3 Results

### 3.1 Causal effects of autoimmune diseases on pancreatitis

A total of 90 SNPs related to RA, 45 SNPs related to SLE, 117 SNPs related to IBD, 89 SNPs related to CD, 62 SNPs related to UC, and 37 SNPs related to T1D were identified, serving as IVs for autoimmune diseases (Tables S2–S7). The *F* statistics for IVs of RA, SLE, IBD, CD, UC, and T1D were >10.

**Figure 2:**
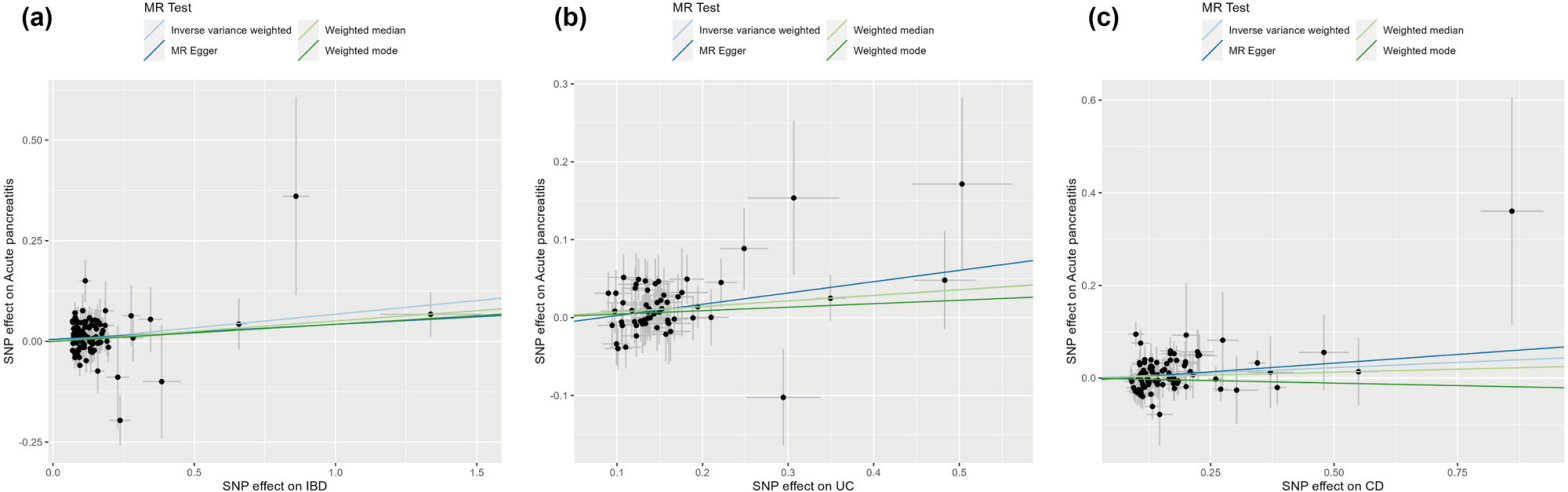
Scatter plots of MR analysis: (a) IBD on acute pancreatitis; (b) UC on acute pancreatitis; (c) CD on acute pancreatitis. The slope of each line represents the causal effect estimated by IVW, MR Egger, weighted median, weight mode, and simple mode methods. MR, Mendelian randomization; SNPs, single-nucleotide polymorphisms.

**Figure 3:**
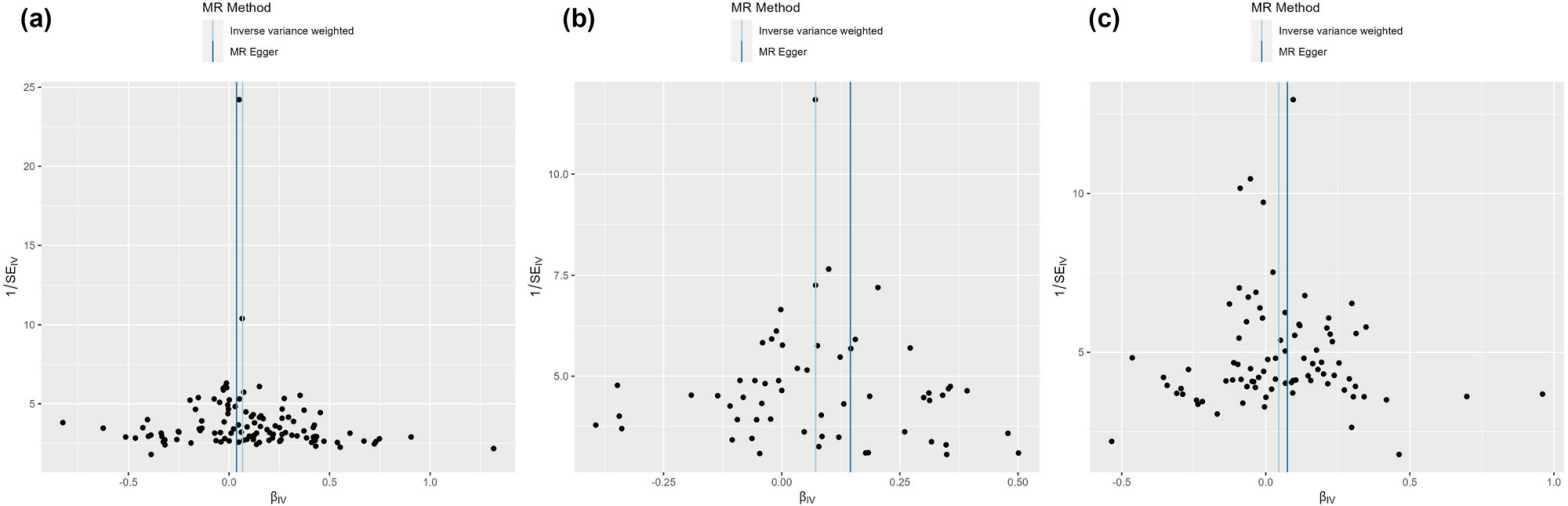
Funnel of MR analysis: (a) IBD on acute pancreatitis; (b) UC on acute pancreatitis; and (c) CD on acute pancreatitis. The symmetry of the funnel plots indicates the absence of heterogeneity among the SNPs.

The MR analysis was conducted with AP, CP, alcohol-induced AP, and alcohol-induced CP as outcomes (Table 1). The IVW analysis indicated that IBD was a risk factor for the incidence of AP (OR = 1.07, 95% CI = 1.03–1.12, *P* = 0.0015). In addition, subgroup analysis showed that UC and CD were potential risk factors for the incidence of AP (UC: OR = 1.07, 95% CI = 1.02–1.13, *P* = 0.01; CD: OR = 1.05, 95% CI = 1–1.09, *P* = 0.03). The other three methods, including MR-Egger, weighted median, and weighted mode methods, showed no statistical significance (Table 1 and Figure 2). The effect size of SNPs is shown in the scatter plot and forest plot (Figures 2 and 3). In addition, no genetically causal association of RA, SLE, IBD, CD, UC, and T1D on CP, alcohol-induced AP, or alcohol-induced CP was found.

**Table 1:** The causal relationship between four pancreatitis exposures and six autoimmune diseases as outcomes using MR

Outcome	Exposure	N. SNPs	Methods	OR (95% CI)	*P*
Acute pancreatitis	RA	85	IVW	1 (0.95–1.04)	0.90
			MR Egger	0.95 (0.89–1.01)	0.13
			Weighted median	1.01 (0.94–1.08)	0.77
			Weighted mode	0.97 (0.91–1.02)	0.24
	SLE	44	IVW	1 (0.97–1.03)	0.98
			MR Egger	0.98 (0.91–1.05)	0.54
			Weighted median	1 (0.95–1.04)	0.90
			Weighted mode	1.01 (0.95–1.08)	0.71
	IBD	113	IVW	1.07 (1.03–1.12)	0.0015
			MR Egger	1.04 (0.96–1.12)	0.32
			Weighted median	1.05 (0.97–1.14)	0.22
			Weighted mode	1.04 (0.98–1.12)	0.22
	CD	84	IVW	1.05 (1–1.09)	0.03
			MR Egger	1.08 (0.96–1.21)	0.19
			Weighted median	1.03 (0.96–1.09)	0.43
			Weighted mode	0.98 (0.87–1.1)	0.71
	UC	58	IVW	1.07 (1.02–1.13)	0.01
			MR Egger	1.16 (0.99–1.36)	0.08
			Weighted median	1.07 (0.99–1.16)	0.08
			Weighted mode	1.05 (0.93–1.18)	0.46
	T1D	34	IVW	1.01 (0.99–1.03)	0.37
			MR Egger	1.01 (0.98–1.05)	0.39
			Weighted median	1.02 (0.99–1.06)	0.15
			Weighted mode	1.02 (0.99–1.04)	0.24
Chronic pancreatitis	RA	85	IVW	1.02 (0.96–1.08)	0.48
			MR Egger	1.03 (0.94–1.12)	0.58
			Weighted median	1.08 (0.99–1.18)	0.07
			Weighted mode	1.07 (0.99–1.16)	0.09
	SLE	44	IVW	0.99 (0.95–1.03)	0.53
			MR Egger	0.97 (0.89–1.07)	0.57
			Weighted median	0.98 (0.93–1.04)	0.60
			Weighted mode	0.97 (0.91–1.04)	0.38
	IBD	113	IVW	1.01 (0.96–1.07)	0.63
			MR Egger	0.89 (0.81–0.98)	0.02
			Weighted median	0.93 (0.84–1.01)	0.10
			Weighted mode	0.94 (0.85–1.04)	0.21
	CD	84	IVW	1.06 (1–1.11)	0.05
			MR Egger	0.98 (0.85–1.14)	0.80
			Weighted median	1.03 (0.95–1.13)	0.43
			Weighted mode	1.03 (0.92–1.16)	0.59
	UC	58	IVW	1.01 (0.94–1.08)	0.76
			MR Egger	0.8 (0.65–0.99)	0.04
			Weighted median	0.94 (0.85–1.05)	0.28
			Weighted mode	0.92 (0.78–1.1)	0.38
	T1D	34	IVW	1.01 (0.97–1.04)	0.68
			MR Egger	1.01 (0.96–1.06)	0.79
			Weighted median	1.04 (1–1.08)	0.07
			Weighted mode	1.03 (1–1.07)	0.10
Alcohol-induced acute pancreatitis	RA	85	IVW	0.99 (0.89–1.09)	0.78
			MR Egger	0.91 (0.77–1.06)	0.23
			Weighted median	0.93 (0.78–1.1)	0.40
			Weighted mode	0.9 (0.78–1.04)	0.15
	SLE	44	IVW	0.95 (0.88–1.02)	0.17
			MR Egger	0.97 (0.82–1.15)	0.73
			Weighted median	0.92 (0.82–1.03)	0.16
			Weighted mode	0.91 (0.78–1.06)	0.22
	IBD	113	IVW	1.02 (0.91–1.13)	0.76
			MR Egger	1.02 (0.85–1.23)	0.81
			Weighted median	1 (0.82–1.22)	0.99
			Weighted mode	1.02 (0.85–1.22)	0.86
	CD	84	IVW	1 (0.9–1.11)	0.94
			MR Egger	1.15 (0.86–1.52)	0.35
			Weighted median	0.99 (0.84–1.15)	0.85
			Weighted mode	0.99 (0.79–1.25)	0.94
	UC	58	IVW	1.11 (0.97–1.26)	0.14
			MR Egger	1.29 (0.86–1.93)	0.22
			Weighted median	1.15 (0.94–1.4)	0.18
			Weighted mode	1.2 (0.87–1.64)	0.27
	T1D	34	IVW	0.96 (0.91–1.02)	0.20
			MR Egger	0.96 (0.89–1.04)	0.31
			Weighted median	0.98 (0.91–1.06)	0.67
			Weighted mode	0.97 (0.91–1.03)	0.31
	RA	85	IVW	0.98 (0.91–1.05)	0.59
			MR Egger	0.97 (0.87–1.08)	0.59
			Weighted median	1 (0.89–1.11)	0.94
			Weighted mode	0.98 (0.89–1.08)	0.73
	SLE	44	IVW	0.97 (0.92–1.02)	0.25
			MR Egger	0.97 (0.86–1.08)	0.56
			Weighted median	1 (0.92–1.08)	0.97
			Weighted mode	1.02 (0.92–1.13)	0.71
	IBD	113	IVW	0.99 (0.92–1.07)	0.81
			MR Egger	0.9 (0.79–1.03)	0.13
			Weighted median	1 (0.88–1.15)	0.94
			Weighted mode	0.96 (0.85–1.08)	0.49
	CD	84	IVW	1.03 (0.95–1.12)	0.43
			MR Egger	0.9 (0.73–1.12)	0.36
			Weighted median	1 (0.9–1.11)	0.99
			Weighted mode	0.96 (0.81–1.12)	0.59
	UC	58	IVW	0.95 (0.86–1.04)	0.23
			MR Egger	0.7 (0.53–0.92)	0.01
			Weighted median	0.87 (0.75–0.99)	0.04
			Weighted mode	0.85 (0.69–1.05)	0.15
	T1D	34	IVW	0.96 (0.92–1.01)	0.09
			MR Egger	0.97 (0.91–1.03)	0.38
			Weighted median	1 (0.94–1.05)	0.91
			Weighted mode	0.98 (0.93–1.03)	0.49
RA	Acute pancreatitis	6	IVW	1.1 (0.98–1.24)	0.10
			MR Egger	1.24 (1–1.53)	0.12
			Weighted median	1.09 (0.95–1.26)	0.23
			Weighted mode	1.1 (0.9–1.34)	0.40
	Chronic pancreatitis	12	IVW	1 (0.95–1.05)	0.98
			MR Egger	0.94 (0.82–1.08)	0.43
			Weighted median	0.98 (0.92–1.05)	0.52
			Weighted mode	0.96 (0.87–1.06)	0.45
	Alcohol-induced acute pancreatitis	7	IVW	1.02 (0.99–1.05)	0.23
			MR Egger	1 (0.96–1.05)	0.97
			Weighted median	1.01 (0.97–1.04)	0.76
			Weighted mode	1 (0.97–1.04)	0.80
	Alcohol-induced chronic pancreatitis	9	IVW	1.01 (0.95–1.07)	0.83
			MR Egger	1.15 (0.99–1.33)	0.12
			Weighted median	0.98 (0.92–1.05)	0.58
			Weighted mode	0.97 (0.89–1.07)	0.57
SLE	Acute pancreatitis	6	IVW	1.06 (0.89–1.25)	0.53
			MR Egger	1.05 (0.76–1.45)	0.77
			Weighted median	1.04 (0.84–1.3)	0.72
			Weighted mode	1.05 (0.77–1.44)	0.75
	Chronic pancreatitis	7	IVW	1.11 (0.95–1.3)	0.19
			MR Egger	1.34 (0.83–2.16)	0.28
			Weighted median	1.17 (0.98–1.39)	0.08
			Weighted mode	1.18 (0.92–1.51)	0.23
	Alcohol-induced acute pancreatitis	7	IVW	1 (0.94–1.06)	0.97
			MR Egger	1 (0.9–1.1)	0.97
			Weighted median	1 (0.93–1.08)	0.94
			Weighted mode	1 (0.93–1.08)	0.91
	Alcohol-induced chronic pancreatitis	6	IVW	0.96 (0.86–1.07)	0.47
			MR Egger	1.05 (0.84–1.3)	0.70
			Weighted median	0.91 (0.78–1.06)	0.22
			Weighted mode	0.89 (0.71–1.12)	0.35
IBD	Acute pancreatitis	8	IVW	0.98 (0.92–1.04)	0.51
			MR Egger	0.94 (0.85–1.04)	0.25
			Weighted median	0.97 (0.9–1.06)	0.55
			Weighted mode	0.92 (0.82–1.03)	0.18
	Chronic pancreatitis	10	IVW	1.01 (0.96–1.05)	0.78
			MR Egger	1.11 (0.98–1.26)	0.15
			Weighted median	1.03 (0.97–1.09)	0.35
			Weighted mode	1.05 (0.95–1.16)	0.38
	Alcohol-induced acute pancreatitis	8	IVW	0.97 (0.95–1)	0.09
			MR Egger	0.99 (0.95–1.04)	0.72
			Weighted median	0.98 (0.95–1.01)	0.12
			Weighted mode	0.98 (0.96–1.01)	0.22
	Alcohol-induced chronic pancreatitis	6	IVW	1.03 (0.99–1.07)	0.20
			MR Egger	1.08 (0.99–1.18)	0.15
			Weighted median	1.01 (0.96–1.07)	0.67
			Weighted mode	1 (0.94–1.08)	0.93
CD	Acute pancreatitis	8	IVW	0.96 (0.89–1.04)	0.31
			MR Egger	0.96 (0.85–1.09)	0.57
			Weighted median	0.98 (0.89–1.08)	0.63
			Weighted mode	0.97 (0.87–1.09)	0.66
	Chronic pancreatitis	10	IVW	1 (0.94–1.06)	0.96
			MR Egger	1.05 (0.89–1.23)	0.60
			Weighted median	1.01 (0.93–1.09)	0.89
			Weighted mode	1.04 (0.93–1.17)	0.52
	Alcohol-induced acute pancreatitis	8	IVW	0.97 (0.94–1)	0.05
			MR Egger	0.99 (0.95–1.04)	0.76
			Weighted median	0.98 (0.95–1.01)	0.29
			Weighted mode	0.99 (0.96–1.02)	0.49
	Alcohol-induced chronic pancreatitis	6	IVW	1.03 (0.96–1.1)	0.43
			MR Egger	1.1 (0.96–1.25)	0.25
			Weighted median	1 (0.93–1.08)	0.99
			Weighted mode	0.99 (0.92–1.08)	0.90
UC	Acute pancreatitis	8	IVW	0.98 (0.9–1.07)	0.61
			MR Egger	0.92 (0.8–1.06)	0.28
			Weighted median	0.94 (0.85–1.05)	0.29
			Weighted mode	0.91 (0.78–1.05)	0.22
	Chronic pancreatitis	10	IVW	0.99 (0.94–1.05)	0.77
			MR Egger	1.1 (0.93–1.29)	0.29
			Weighted median	1.02 (0.95–1.11)	0.55
			Weighted mode	1.04 (0.92–1.18)	0.51
	Alcohol-induced acute pancreatitis	8	IVW	0.98 (0.95–1.01)	0.21
			MR Egger	0.98 (0.93–1.04)	0.56
			Weighted median	0.97 (0.94–1)	0.09
			Weighted mode	0.97 (0.93–1)	0.10
	Alcohol-induced chronic pancreatitis	6	IVW	1.02 (0.97–1.08)	0.45
			MR Egger	1.06 (0.95–1.19)	0.33
			Weighted median	1.04 (0.97–1.11)	0.33
			Weighted mode	1.05 (0.96–1.16)	0.34
T1D	Acute pancreatitis	8	IVW	1.02 (0.89–1.18)	0.74
			MR Egger	1.12 (0.83–1.51)	0.51
			Weighted median	1.06 (0.88–1.26)	0.56
			Weighted mode	1.07 (0.86–1.33)	0.58
	Chronic pancreatitis	10	IVW	1.01 (0.93–1.09)	0.90
			MR Egger	1.04 (0.83–1.31)	0.71
			Weighted median	1.01 (0.91–1.12)	0.82
			Weighted mode	1.02 (0.87–1.18)	0.85
	Alcohol-induced acute pancreatitis	8	IVW	0.97 (0.94–1.01)	0.15
			MR Egger	1 (0.95–1.05)	0.94
			Weighted median	0.98 (0.94–1.03)	0.48
			Weighted mode	0.98 (0.94–1.03)	0.45
	Alcohol-induced chronic pancreatitis	6	IVW	1.04 (0.97–1.13)	0.27
			MR Egger	1.03 (0.87–1.21)	0.75
			Weighted median	1.03 (0.93–1.14)	0.57
			Weighted mode	1.04 (0.91–1.19)	0.62

Note: RA: rheumatoid arthritis; SLE: systemic lupus erythematosus; IBD: inflammatory bowel disease; CD: Crohn’s disease; UC: ulcerative colitis; T1D: type 1 diabetes.

**Figure 4:**
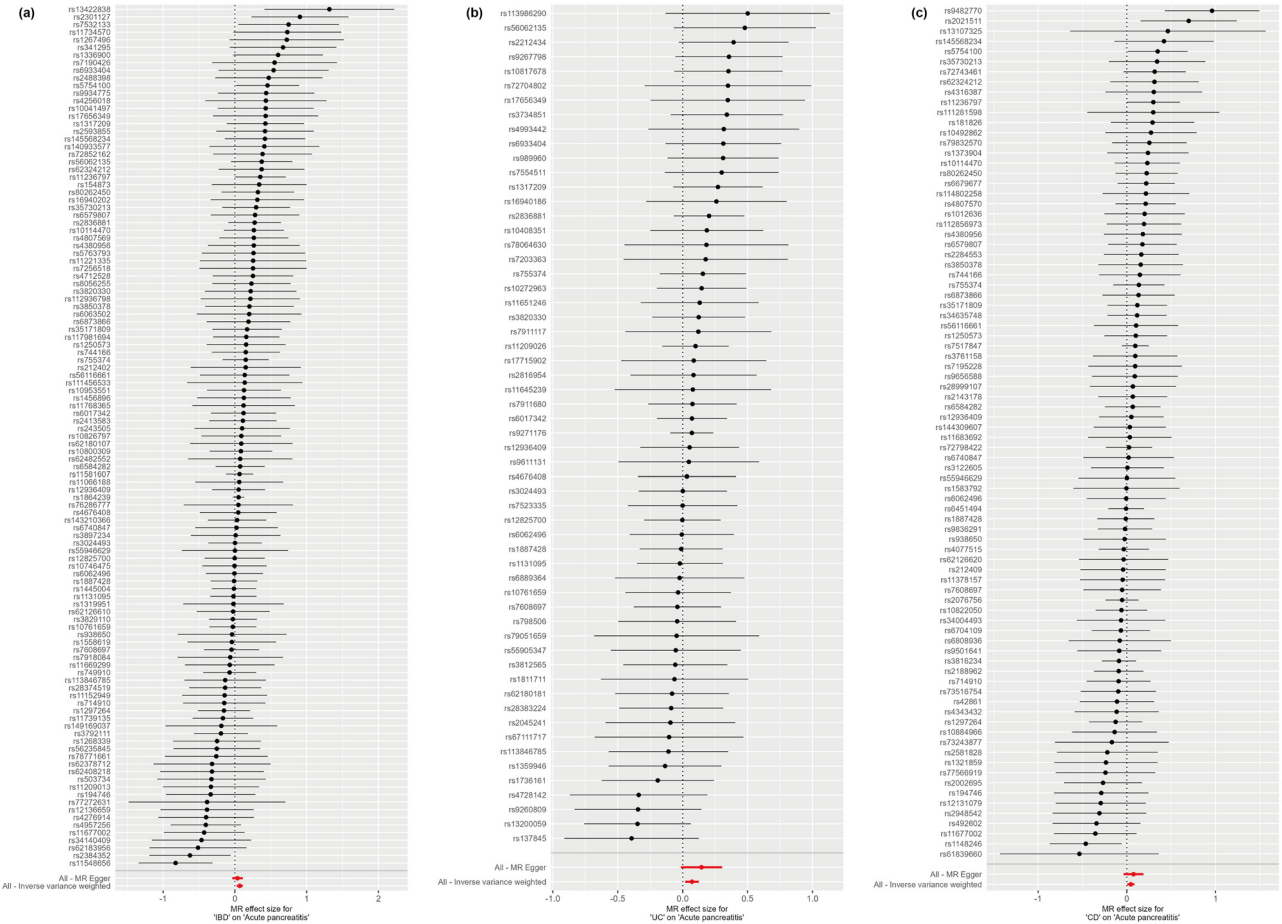
Forest of MR analysis: (a) IBD on acute pancreatitis; (b) UC on acute pancreatitis; and (c) CD on acute pancreatitis. The forest plots show each SNP MR estimate and 95% CI values (gray line segment). CI, confidence interval; IVW, inverse variance weighted; MR, Mendelian randomization; SNPs, single-nucleotide polymorphisms.

**Figure 5:**
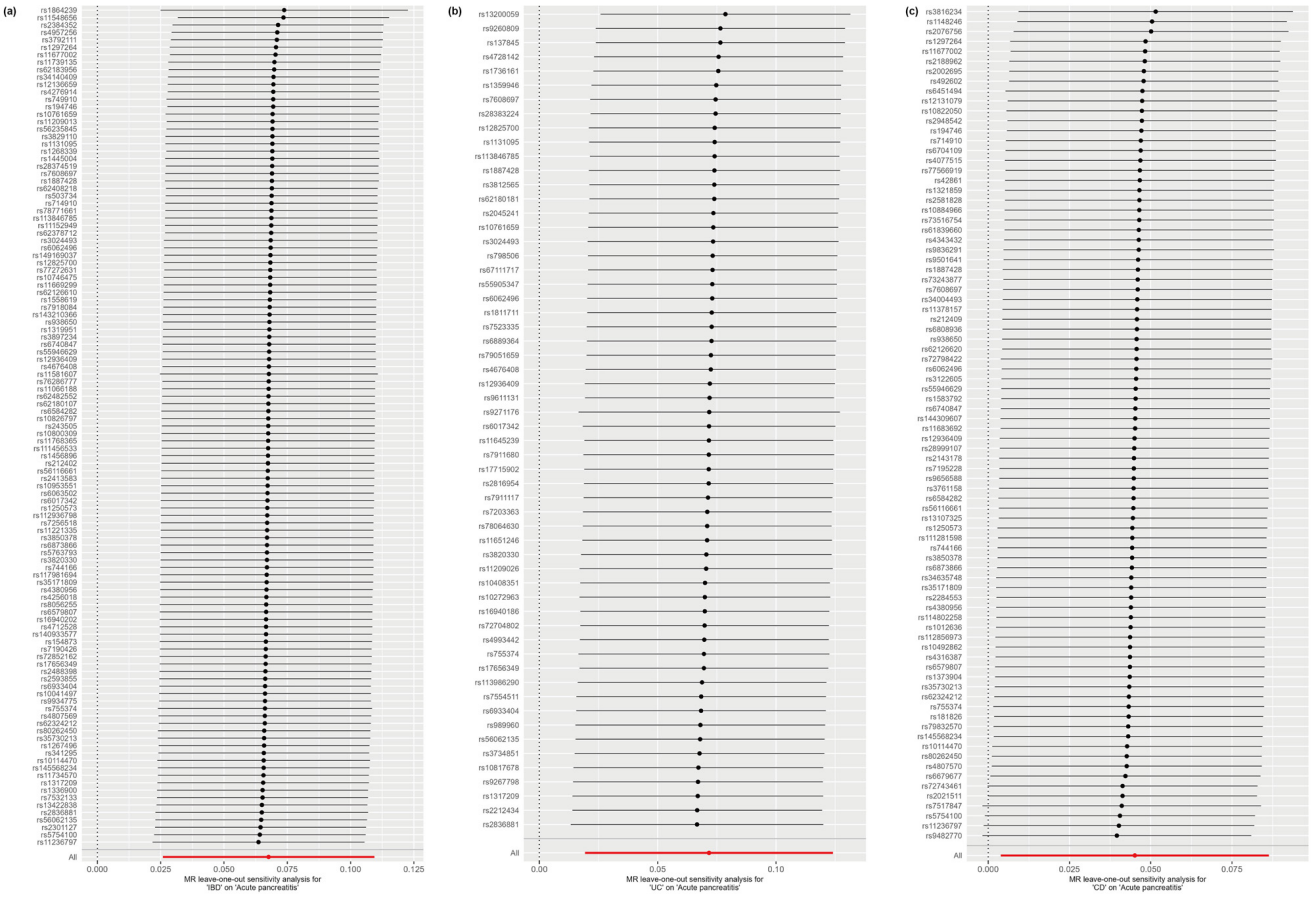
Leave-one-out analysis of MR analysis. The leave-one-out plots show the MR estimate and 95% CI values after removing the corresponding single SNP. (a) IBD on acute pancreatitis; (b) UC on acute pancreatitis; and (c) CD on acute pancreatitis.

Cochran’s *Q* test and MR-Egger regression revealed no heterogeneity and horizontal pleiotropy for IBD, UC, and CD in CP (*P* > 0.05). Leave-one-out analysis indicated that the causal estimates of IBD, UC, and CD and subtypes were not driven by any single SNP. The leave-one-out analysis plots, forest plots, and funnel plots are shown in Figures 3–5. Moreover, there was no heterogeneity between the individual SNP in most other autoimmune diseases but heterogeneity for T1D in CP (*Q* = 49.67; *P* = 0.03). The MR-Egger regression results indicated that the analyses of CP and IBD (*P* = 0.002), alcohol-induced AP and UC (*P* = 0.028), and alcohol-induced CP and UC (*P* = 0.03) were affected by horizontal pleiotropy (Table 2). However, the leave-one-out and MR-PRESSO did not show any outliers for these three pairs of exposure and outcome (Table 3). MR-PRESSO verification of the causal effect of IBD, UC, and CD on AP (IBD: OR = 1.07 95% CI = 1.03–1.11, *P* = 0.002; UC: OR = 1.07 95% CI = 1.02–1.11, *P* = 0.005; CD: OR = 1.04 95% CI = 1.00–1.09, *P* = 0.04) (Table 3).

**Table 2:** Heterogeneity and pleiotropy between four pancreatitis and six autoimmune diseases

Outcome	Exposure	Heterogeneity		Pleiotropy	
		*Q* statistic (IVW)	*P* value	MR-Egger intercept	*P* value
Acute pancreatitis	RA	102.38	0.08	0.01	0.06
	SLE	44.95	0.39	0.01	0.48
	IBD	113.06	0.45	0.01	0.33
	CD	76.56	0.68	−0.01	0.58
	UC	40.85	0.95	−0.01	0.34
	T1D	36.17	0.32	−0.002	0.77
Chronic pancreatitis	RA	105.21	0.06	0.008	0.90
	SLE	44.55	0.41	0.005	0.75
	IBD	112.25	0.48	0.021	0.002
	CD	67.79	0.89	0.01	0.30
	UC	45.50	0.86	0.04	0.03
	T1D	49.67	0.03	0.0007	0.96
Alcohol-induced acute pancreatitis	RA	90.58	0.29	0.019	0.18
	SLE	35.87	0.77	−0.009	0.76
	IBD	114.92	0.41	−0.001	0.93
	CD	79.69	0.58	−0.022	0.33
	UC	44.34	0.89	−0.025	0.43
	T1D	26.84	0.77	0.002	0.92
Alcohol-induced chronic pancreatitis	RA	85.13	0.46	0.002	0.80
	SLE	39.18	0.64	0.002	0.94
	IBD	127.28	0.15	0.016	0.09
	CD	101.73	0.08	0.023	0.20
	UC	49.72	0.74	0.049	0.028
	T1D	46.53	0.06	−0.009	0.57
RA	Acute pancreatitis	6.38	0.27	−0.025	0.28
	Chronic pancreatitis	10.78	0.46	0.012	0.40
	Alcohol-induced acute pancreatitis	2.17	0.91	0.010	0.44
	Alcohol-induced chronic pancreatitis	11.17	0.19	−0.038	0.12
SLE	Acute pancreatitis	4.19	0.52	8.72 × 10^−4^	0.97
	Chronic pancreatitis	9.48	0.15	−0.04	0.45
	Alcohol-induced acute pancreatitis	6.95	0.33	3.71 × 10^−4^	0.99
	Alcohol-induced chronic pancreatitis	4.13	0.53	−0.033	0.41
IBD	Acute pancreatitis	7.60	0.37	0.013	0.32
	Chronic pancreatitis	6.32	0.71	−0.02	0.15
	Alcohol-induced acute pancreatitis	16.53	0.02	−0.017	0.37
	Alcohol-induced chronic pancreatitis	5.06	0.41	−0.02	0.27
CD	Acute pancreatitis	2.96	0.89	−4.31 × 10^−4^	0.98
	Chronic pancreatitis	5.69	0.77	−0.009	0.59
	Alcohol-induced acute pancreatitis	11.09	0.13	−0.024	0.18
	Alcohol-induced chronic pancreatitis	7.44	0.19	−0.027	0.34
UC	Acute pancreatitis	8.83	0.27	0.02	0.31
	Chronic pancreatitis	6.17	0.72	−0.02	0.22
	Alcohol-induced acute pancreatitis	12.32	0.09	−0.004	0.82
	Alcohol-induced chronic pancreatitis	3.07	0.69	−0.017	0.45
T1D	Acute pancreatitis	1.83	0.77	−0.02	0.55
	Chronic pancreatitis	3.96	0.91	−0.008	0.73
	Alcohol-induced acute pancreatitis	7.76	0.35	−0.028	0.18
	Alcohol-induced chronic pancreatitis	4.62	0.46	0.006	0.85

Note: RA: rheumatoid arthritis; SLE: systemic lupus erythematosus; IBD: inflammatory bowel disease; CD: Crohn’s disease; UC: ulcerative colitis; T1D: type 1 diabetes.

**Table 3:** Testing Pleiotropy of four pancreatitis and six autoimmune diseases using MRPRESSO

Exposure	Outcome	Raw		Outlier corrected		Global *P*	Number of outliers	Distortion *P*
		OR (CI%)	*P*	OR (CI%)	*P*			
RA	Acute pancreatitis	1 (0.95–1.04)	0.91	NA	NA	0.09	NA	NA
SLE	Acute pancreatitis	1 (0.97–1.03)	0.94	NA	NA	0.38	NA	NA
IBD	Acute pancreatitis	1.07 (1.03–1.11)	0.002	NA	NA	0.50	NA	NA
CD	Acute pancreatitis	1.04 (1.00–1.09)	0.04	NA	NA	0.65	NA	NA
UC	Acute pancreatitis	1.07 (1.02–1.11)	0.005	NA	NA	0.97	NA	NA
T1D	Acute pancreatitis	1.01 (0.99–1.04)	0.28	NA	NA	0.19	NA	NA
RA	Chronic pancreatitis	1.02 (0.96–1.08)	0.58	NA	NA	0.04	NA	NA
SLE	Chronic pancreatitis	0.99 (0.95–1.03)	0.54	NA	NA	0.45	NA	NA
IBD	Chronic pancreatitis	1.01 (0.96–1.07)	0.68	NA	NA	0.45	NA	NA
CD	Chronic pancreatitis	1.05 (1–1.11)	0.03	NA	NA	0.92	NA	NA
UC	Chronic pancreatitis	1 (0.94–1.06)	0.91	NA	NA	0.89	NA	NA
T1D	Chronic pancreatitis	1.01 (0.97–1.05)	0.61	NA	NA	0.05	NA	NA
RA	Alcohol-induced acute pancreatitis	0.98 (0.88–1.09)	0.71	NA	NA	0.29	NA	NA
SLE	Alcohol-induced acute pancreatitis	0.95 (0.88–1.02)	0.13	NA	NA	0.80	NA	NA
IBD	Alcohol-induced acute pancreatitis	1.01 (0.91–1.12)	0.86	NA	NA	0.39	NA	NA
CD	Alcohol-induced acute pancreatitis	1.01 (0.91–1.11)	0.90	NA	NA	0.64	NA	NA
UC	Alcohol-induced acute pancreatitis	1.09 (0.97–1.22)	0.16	NA	NA	0.87	NA	NA
T1D	Alcohol-induced acute pancreatitis	0.97 (0.92–1.01)	0.17	NA	NA	0.78	NA	NA
RA	Alcohol-induced chronic pancreatitis	0.98 (0.91–1.05)	0.56	NA	NA	0.47	NA	NA
SLE	Alcohol-induced chronic pancreatitis	0.97 (0.92–1.02)	0.25	NA	NA	0.65	NA	NA
IBD	Alcohol-induced chronic pancreatitis	0.99 (0.92–1.07)	0.79	NA	NA	0.19	NA	NA
CD	Alcohol-induced chronic pancreatitis	1.04 (0.96–1.12)	0.36	NA	NA	0.084	NA	NA
UC	Alcohol-induced chronic pancreatitis	0.94 (0.87–1.02)	0.15	NA	NA	0.81	NA	NA
T1D	Alcohol-induced chronic pancreatitis	0.96 (0.92–1.01)	0.12	NA	NA	0.08	NA	NA
Acute pancreatitis	RA	1.08 (0.98–1.20)	0.17	NA	NA	0.36	NA	NA
Chronic pancreatitis	RA	1 (0.95–1.05)	0.98	NA	NA	0.46	NA	NA
Alcohol-induced acute pancreatitis	RA	1.01 (1–1.03)	0.13	NA	NA	0.92	NA	NA
Alcohol-induced chronic pancreatitis	RA	1.01 (0.95–1.07)	0.83	NA	NA	0.22	NA	NA
Acute pancreatitis	SLE	1 (0.86–1.15)	0.96	NA	NA	0.36	NA	NA
Chronic pancreatitis	SLE	1.11 (0.99–1.25)	0.12	NA	NA	0.32	NA	NA
Alcohol-induced acute pancreatitis	SLE	1 (0.94–1.06)	0.99	NA	NA	0.29	NA	NA
Alcohol-induced chronic pancreatitis	SLE	1.01 (0.92–1.09)	0.91	NA	NA	0.49	NA	NA
Acute pancreatitis	IBD	0.97 (0.91–1.03)	0.34	NA	NA	0.33	NA	NA
Chronic pancreatitis	IBD	1.02 (0.98–1.05)	0.32	NA	NA	0.74	NA	NA
Alcohol-induced acute pancreatitis	IBD	0.98 (0.95–1.01)	0.18	NA	NA	0.06	NA	NA
Alcohol-induced chronic pancreatitis	IBD	1.03 (1–1.06)	0.13	NA	NA	0.62	NA	NA
Acute pancreatitis	CD	0.95 (0.91–0.99)	0.05	NA	NA	0.96	NA	NA
Chronic pancreatitis	CD	1.02 (0.97–1.08)	0.40	NA	NA	0.49	NA	NA
Alcohol-induced acute pancreatitis	CD	0.98 (0.95–1.01)	0.16	NA	NA	0.19	NA	NA
Alcohol-induced chronic pancreatitis	CD	1.03 (0.98–1.08)	0.25	NA	NA	0.46	NA	NA
Acute pancreatitis	UC	0.97 (0.88–1.06)	0.52	NA	NA	0.07	NA	NA
Chronic pancreatitis	UC	0.99 (0.96–1.04)	0.80	NA	NA	0.81	NA	NA
Alcohol-induced acute pancreatitis	UC	0.98 (0.95–1.01)	0.19	NA	NA	0.29	NA	NA
Alcohol-induced chronic pancreatitis	UC	1.02 (0.99–1.06)	0.29	NA	NA	0.81	NA	NA
Acute pancreatitis	T1D	1.01 (0.94–1.1)	0.76	NA	NA	0.85	NA	NA
Chronic pancreatitis	T1D	1.01 (0.97–1.05)	0.63	NA	NA	0.99	NA	NA
Alcohol-induced acute pancreatitis	T1D	0.97 (0.94–1.01)	0.15	NA	NA	0.47	NA	NA
Alcohol-induced chronic pancreatitis	T1D	1.02 (0.96–1.08)	0.59	NA	NA	0.56	NA	NA

### 3.2 Causal effects of pancreatitis on autoimmune diseases

A total of 11 SNPs related to AP, 14 SNPs related to CP, 11 SNPs related to alcohol-induced AP, and 10 SNPs related to alcohol-induced CP were identified. All the *F*-statistic values were >10 (Tables S8–S11).

The MR analysis was conducted with RA, SLE, IBD, CD, UC, and T1D as outcomes (Table 1). The IVW analysis showed no significant association causal relationship between these six types of autoimmune disease risks and AP, CP, alcohol-induced AP, or alcohol-induced CP. Further MR analyses, including MR-Egger analysis, weighted median analysis, and weighted mode analysis, also detected no significant association between autoimmune disease risks and pancreatitis (all *P* > 0.05).

The Cochrane *Q* statistics in IVW and MR-Egger methods disclosed no marked heterogeneity (*P* > 0.05), and the MR-Egger regression showed an absence of pleiotropy (*P* > 0.05) (Table 2). To affirm the reliability of these conclusions, a “leave-one-out” sensitivity test was conducted, revealing that the causal association was not reliant on any single SNP. The MR-PRESSO test uncovered no evidence of horizontal pleiotropy (Table 3).

## 4 Discussion

The present study used a two-sample MR analysis to evaluate the potential causal relationships between AP/CP and six autoimmune diseases. Our findings revealed a significant genetic correlation between IBD and the risk of developing AP, as well as UC and AP, CD, and AP, However, no genetically causal relationships were found between other autoimmune diseases (RA, SLE, and T1D) and pancreatitis. The reverse MR analysis did not identify any causal links from pancreatitis to these autoimmune diseases. Sensitivity analyses further solidified the robustness of these discoveries.

Furthermore, a study based on 4,223 patients with IBD-associated AP suggested a strong association between IBD medications such as thiopurines, 6-mercaptopurine, and 5-aminosalicylic acid with the incidence of AP [20]. Notably, the increased risk of AP associated with thiopurine use was notably higher than that in UC. Thiopurine has also been recognized as a key trigger for AP, with approximately 5% of IBD patients developing AP [21]. These findings highlight that medications used in the treatment of IBD can also elevate the risk of AP. These effects may affect the effect of IBD on pancreatitis in observational studies. A recent meta-analysis reported that patients with IBD have an increased risk of developing AP (hazard ratio [HR]: 2.78, 95% CI: 2.40–3.22), with CD (HR: 3.62, 95% CI: 2.99–4.38) having a higher risk than UC (HR: 2.24, 95% CI: 1.85–2.71) [22]. Our study identified a similar trend; however, we did not observe a significantly higher risk of CD compared to UC. This discrepancy may be due to differences in common treatment approaches for these conditions. Moreover, a cohort time analysis indicates that within the first year following an IBD diagnosis, 85% of pediatric IBD patients and 69% of adult IBD patients develop pancreatitis and pancreatitis occurs most commonly at the time of the initial IBD diagnosis, with an incidence rate ranging from 9.3 to 16.2% across all IBD cohorts [23]. These findings suggest that pancreatitis in IBD patients may not solely be a consequence of medication side effects but might also be directly influenced by the adverse effects of intestinal inflammation on the pancreas. Integrating these insights with our results suggests that there should be a heightened vigilance for AP from the onset of IBD diagnosis, with careful use of medications to mitigate the increased risk.

The association between IBD and AP is multifaceted, involving shared inflammatory pathways, the gut–pancreas axis, medication-related impacts, and gut microbiome dysbiosis. Both conditions may share pro-inflammatory cytokines, such as IL-33 and TNF-α, which intensify inflammation and predispose individuals to pancreatitis [24]. The anatomical and functional relationship known as the gut–pancreas axis further facilitates the spread of inflammation from the gastrointestinal tract to the pancreas, enhancing localized immune responses [25]. Additionally, medications used in IBD treatment, like immunosuppressants and anti-inflammatory drugs including thiopurines and 5-aminosalicylic acid, can increase pancreatitis risk due to adverse effects [26]. Alterations in the gut microbiome, a common occurrence in IBD, also contribute to pancreatitis by disrupting immune regulation and increasing intestinal permeability [27]. Our research emphasizes that these interactions predominantly increase the risk of AP, triggered by rapid shifts in gut microbiota or acute cytokine release, distinguishing it from chronic forms of the disease. Although our study identifies a genetic causal relationship between IBD and pancreatitis, it is important to note that in clinical practice, pancreatitis may be drug-induced. Clinicians must differentiate whether pancreatitis is caused by medications or is a direct manifestation of IBD itself, to implement personalized medical treatment. This complex interplay underscores the critical need for careful management and monitoring of IBD to mitigate the risk of developing acute pancreatitis.

Although we did not detect a causal relationship between acute/CP and RA, SLE, or T1D, multiple studies have suggested a high correlation between the diseases. A cohort study analyzing 29,755 RA patients in Taiwan found that RA patients had a higher risk of AP with an HR of 1.62 (95% CI: 1.43–1.83). Oral corticosteroids were found to reduce the risk of AP (HR: 0.83, 95% CI: 0.73–0.94), whereas antirheumatic drugs or tumor necrosis factor blockers did not reduce the risk [28]. A recent study based on a multicenter database in the United States (including 518,280 RA patients) found that RA patients were more likely to develop AP (OR: 2.51; 95% CI: 2.41–2.60) and CP (OR: 2.97; 95% CI: 2.70–3.26) [6]. Observational cohorts have confirmed that SLE patients can develop AP, which is closely related to the activity level of SLE [29]. An independent cohort study found that children with T1D had increased lipopolysaccharide production by intestinal microbiota, exacerbating pancreatic inflammatory responses [30]. Conversely, a meta-analysis showed that T1D could be induced several years after the occurrence of acute or CP [31], suggesting a potential relationship between pancreatitis and T1D.

Previous observational studies have suggested a close linkage between autoimmune disorders such as RA, SLE, CD, UC, IBD, and T1D with the onset of pancreatitis. However, these diseases employ distinct immune pathways which may not impact the pancreas directly in the same manner. Genetic factors associated with these autoimmune diseases might not significantly overlap with those influencing the development of pancreatitis, explaining the absence of observed causal relationships in our findings. Furthermore, we engaged genetic instruments specifically tied to AP, CP, and their alcohol-induced counterparts for MR analysis. The outcomes divulged no causal correlation between autoimmune maladies and alcohol-driven pancreatitis manifestations. Existing studies suggest that while alcohol exacerbates systemic inflammation, thereby aggravating both acute and chronic forms of pancreatitis and acting as a risk element for autoimmune diseases [32], its impact on pancreatitis may not be mediated directly through autoimmune pathways. Furthermore, alcohol consumption has been flagged as a protective agent against autoimmune conditions like RA and SLE, with animal models demonstrating the capacity of alcohol exposure to modulate helper T cell functionality, leading to induced immune tolerance [33,34]. Although our findings did not detect a genetically causal association between the six autoimmune diseases and alcohol-induced pancreatitis, the extent to which alcohol exposure through autoimmune disease-mediated disruption in immune activity exacerbates pancreatitis severity remains an avenue for future experimental inquiry.

The present study has several advantages. First, it is the first study to utilize MR to explore the genetic causal relationships between pancreatitis and autoimmune diseases. We procured statistical data on four subtypes of pancreatitis and six autoimmune diseases, facilitating a comprehensive evaluation of the causal relationships between them. Furthermore, using bidirectional MR analysis ensures the inference of bidirectional causal relationships between pancreatitis and autoimmune diseases. However, this study also has some limitations. Primarily, the study population consisted of individuals of European descent, leaving the applicability of these findings to other races open for validation. Additionally, the *P*-value of the screening is 5 × 10^−6^, which may bias the results, but in the present study, the IV *F*-statistics were all >10, minimizing bias as much as possible. Third, there might be an overlap between the populations studied for exposure and outcomes could also somewhat impact the MR analysis.

## 5 Conclusions

Our findings demonstrated a genetically causal effect of IBD on AP. This that pancreatitis in clinical IBD patients may solely be a direct consequence of the inflammatory disease itself. These findings emphasize the need for clinical vigilance regarding pancreatitis in the diagnosis and management of IBD patients, beyond the scope of medication effects alone. The results of this study should be further validated based on larger-scale GWAS summary data, more advanced MR analysis methods, and more genetic instruments.

## Supplementary Material

Supplementary Table
